# Supplementary material for: Dynamically weighted graph neural network for detection of early mild cognitive impairment
Source: PLoS One. 2025 Jun 4;20(6):e0323894. doi: 10.1371/journal.pone.0323894 (PMC12136332; doi:10.1371/journal.pone.0323894)
Supplement: Attachment — Submitted filename: respone-r1.pdf [file pone.0323894.s001.pdf]

Response to reviewers

We wish to thank the associate editor and the anonymous reviewers for their insightful comments and suggestions that led to improvements in the quality of the content and the presentation of our paper (*PONE-D-24-16152R1, "Dynamically weighted graph neural network for detection of early mild cognitive impairment "*).

The manuscript has been fully revised. Please find enclosed our comments on the reviewers' suggestions and comments and how we addressed and fixed all the comments and concerns raised.

Reviewer 1:

Comment 1: I suggest adding a definition of low and high order FC.

Response: Thank you for your helpful suggestion. We will add the following definition of low-order and high-order functional connectivity (FC) networks in the revised manuscript to clarify the concept:

Low-order FC networks refer to the direct temporal correlations between pairs of brain regions, where the functional connectivity is typically calculated based on simple pairwise correlations of resting-state fMRI signals. These networks represent the most immediate and direct interactions between regions of the brain.

High-order FC networks go beyond pairwise correlations and involve higher-order interactions, such as the correlations between multiple brain regions simultaneously. These networks capture more complex patterns of interaction, reflecting the higher-level coordination between different brain areas.

We will ensure that these definitions are added to the manuscript to make the distinction between low-order and high-order FC networks clearer for the readers.

Comment 2: It is not clear if the authors are classifying networks or the control vs. eMCI group.

Response: Thank you for your comment. In this work, we are classifying the control vs. eMCI group based on the features extracted from both low-order and high-order functional connectivity

(FC) networks.

To clarify, the FC networks (both low-order and high-order) are constructed from resting-state fMRI (RS-fMRI) data, and the goal is to use these networks to classify individuals into two groups: the eMCI group (early Mild Cognitive Impairment) and the NC group (Normal Control).

The classification is performed using the features derived from the dynamic FC networks, where the low-order FC network captures simple pairwise correlations between brain regions, and the high-order FC network captures more complex interactions between multiple brain regions simultaneously. These extracted features are then fed into classification algorithms to distinguish between the eMCI and NC groups.

Comment 3: Could the authors further explain how "…the proposed model is simple, effective, and easy to implement" compared to the available techniques they considered?
Response: Thank you for your valuable feedback. We agree that further clarification of the simplicity, effectiveness, and ease of implementation of our proposed model would be helpful.

The simplicity of our proposed model lies in its conceptual framework. Unlike some of the more complex methods in the literature, such as temporal GNN (tGNN) and deep learning-based methods, which involve intricate neural network architectures and require fine-tuning of many hyperparameters, our model leverages the functional connectivity (FC) network weights as the adjacency matrix of the graph neural network (GNN). This direct use of FC network weights simplifies the model structure and reduces the need for additional feature engineering or preprocessing steps, making the model straightforward to implement.

Moreover, the input features for the GNN are derived from the local clustering coefficients of the weighted graph of nodes, which are simple statistical properties of the graph and do not require complicated data transformation or heavy computation. This makes the model not only effective but also highly interpretable. In comparison with the other methods we considered, which typically require complex preprocessing and high-dimensional feature extraction, our model stands out due to its reduced reliance on such steps, making it both simple and efficient for practical implementation.

Comment 4: Why are low order FC and high order FC considered separately and not together? Would this approach still be better compared to other models?
Response: Thank you for the insightful question.

The reason we consider low-order FC and high-order FC separately is due to the distinct nature of the interactions they capture.

Low-order FC captures the direct temporal correlations between pairs of brain regions, providing a snapshot of immediate, direct interactions between brain regions. This type of FC network reflects basic, straightforward connectivity patterns that can provide useful information for detecting abnormalities, especially for diseases like eMCI, where early impairments may show up as

disruptions in simple pairwise correlations.

High-order FC, on the other hand, captures higher-order interactions, such as the correlations between multiple brain regions simultaneously. This network reflects more complex and coordinated patterns of brain activity, which may be more indicative of later stages of cognitive impairment or more subtle disruptions in brain function that may not be captured by low-order correlations alone.

By treating these two types of FC networks separately, we allow the model to capture both the immediate and the complex interactions between brain regions. Combining them directly into a single network could lead to the loss of this crucial distinction and potentially blur the differences between early and more advanced stages of brain dysfunction.

As for whether this approach is still better compared to other models, our experimental results demonstrate that considering low-order and high-order FC networks separately yields superior performance in classifying eMCI vs. NC groups. Specifically, our method outperforms comparison methods (SVM, tGNN, TDL, FCDL) in both low-order and high-order FC networks, as shown in Tables 2 and 3. The separate consideration of these networks allows the model to better capture both simple and complex patterns of brain connectivity, which, as shown in the results, leads to improved classification accuracy and F1-score.

Therefore, while it is possible to combine both low- and high-order FC networks into a single model, our approach of treating them separately appears to be more effective, particularly in distinguishing between the eMCI and NC groups.

Comment 5: Is the proposed model less computationally demanding compared to other approaches?

Response: Thank you for raising this important point. We understand that computational efficiency is a key consideration, and we would like to clarify how our model compares in this regard.

The proposed model is less computationally demanding compared to some of the other methods we considered, such as temporal GNN (tGNN) and deep learning-based approaches. These methods involve multiple layers of neural networks, which can be computationally intensive, especially when working with large datasets or high-dimensional features. In contrast, our model uses a GNN with a relatively simple architecture and the local clustering coefficients as the input features, which significantly reduces the computational load.

Additionally, our model does not require the extensive hyperparameter tuning and optimization steps that are often needed for deep learning models. Since the input features are derived directly from the FC network, the model avoids the need for complex preprocessing or dimensionality reduction, further improving its computational efficiency.

In terms of training time, our approach was observed to have faster convergence compared to deep learning methods, due to the fewer parameters involved and the simpler structure. This makes

the proposed model an attractive option when computational resources or time constraints are a concern.

Reviewer 2:

Comment 1: The caption of Fig. 1 lacks sufficient detail. Additional information is needed to explain the key components of the proposed framework. Additionally, 'RNN' should be replaced with 'LSTM' for consistency with the rest of the paper.

Response: Thank you for your feedback. We have updated the caption of Fig. 1 to provide more detailed information, explaining the key components of the proposed framework. Additionally, "RNN" has been replaced with "LSTM" in the figure to ensure consistency with the rest of the paper.

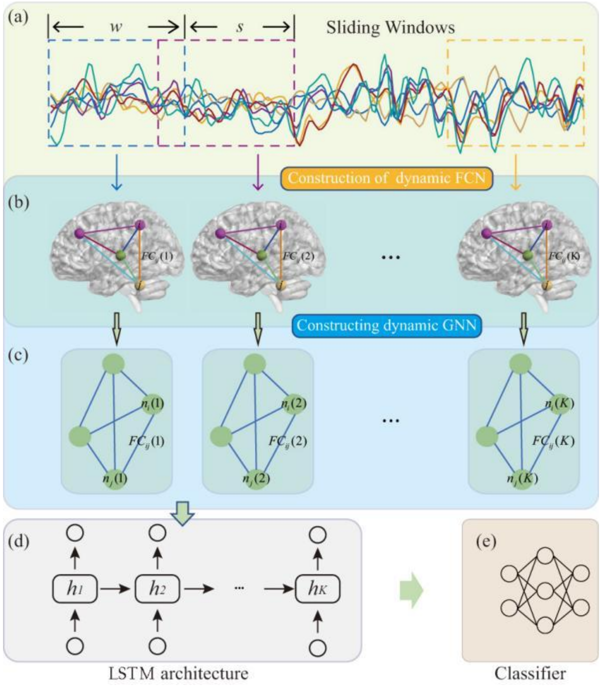

Comment 2: In Eq. (1), the superscript of the first sum in the denominator should be M (not T).

Response: Thank you for pointing that out. We have corrected the superscript of the first sum in the denominator of Eq. (1) to be M, as per your suggestion.

$$lFC_{ij}(k) = \frac{\langle \mathbf{x}_i(k), \mathbf{x}_j(k) \rangle}{|\mathbf{x}_i(k)||\mathbf{x}_j(k)|} = \frac{\sum_{t=1}^{M} (x_{it}(k) - \overline{\mathbf{x}}_i(k))(x_{jt}(k) - \overline{\mathbf{x}}_j(k))}{\sqrt{\sum_{t=1}^{M} (x_{it}(k) - \overline{\mathbf{x}}_i(k))^2} \sqrt{\sum_{t=1}^{M} (x_{jt}(k) - \overline{\mathbf{x}}_j(k))^2}} \quad (1)$$

Comment 3: In Eq. (3), it's better to use the common notation \mathcal{N}_i instead of \Delta_i to denote the set of immediate neighbors of node i.

Response: Thank you for the suggestion. We have updated Eq. (3) to use the common notation \mathcal{N}_i instead of \Delta_i to denote the set of immediate neighbors of node i.

$$n_i(k) = \frac{2\sum_{j:j\in\mathcal{N}_i} (FC_{ij}(k))^{\frac{1}{3}}}{|\mathcal{N}_i|(|\mathcal{N}_i| - 1)} \quad (3)$$

Comment 4: Table 1 is too wide and does not fit on the page, making it unclear if any information is missing.

Response: Thank you for your feedback. We have adjusted the width of Table 1 to ensure it fits within the page, making the information clearer and more readable.

Table 1 Demographic Information of the Subjects

Group	Total Number of Participants	Age (mean $\pm$ SD)	Gender (M/F)	MMSE (mean $\pm$ SD)	APOE $\varepsilon 4$ Carriers (n/N)
NC	154	75.36 ± 6.16	67/87	29.1 ± 1.2	30/154
eMCI	165	72.03 ± 7.26	73/92	27.4 ± 2.1	45/165

Comment 5: The proposed algorithm depends on several hyper-parameters that need to be fine-tuned (e.g., L, R, M, s, and K). So it is unclear how the choice of such hyper-parameters would affect the overall performance.

Response: We appreciate the reviewer’s comment on the hyper-parameters L, R, M, s, and K. The choice of these parameters does indeed impact the performance of the proposed method. Specifically, the window length L and step size s control the trade-off between capturing long-term dependencies and computational efficiency, while the number of brain regions R and the maximum time series length M influence the complexity of the resulting dynamic FC network. The number of windows K, which is derived from L and s, determines the granularity of the temporal dynamic network.

Comment 5: What are the limitations of the proposed approach?

Response: Thank you for the reviewer’s insightful comment. While our proposed method demonstrates promising results, we acknowledge several limitations. First, the method currently focuses on a specific dataset (ADNI) and primarily targets the detection of early MCI (eMCI) as a precursor to Alzheimer's Disease. This limits the generalizability of our approach to other forms of neurodegenerative conditions or different populations. Second, our method relies on the functional connectivity (FC) networks, which are sensitive to various factors, including the quality of the fMRI data, preprocessing techniques, and the choice of brain regions. Variations in these factors may affect the robustness of the model's performance. Lastly, although we integrate topological features with GNN-based methods, further exploration of other dynamic features or more complex models could potentially improve the performance, especially when dealing with heterogeneous data.

In future work, we plan to address these limitations by expanding the dataset to include other subtypes of Alzheimer's Disease and neurodegenerative conditions, as well as improving the model’s ability to handle different forms of data variability and topological characteristics.
