# Supplementary material for: Dynamically weighted graph neural network for detection of early mild cognitive impairment
Source: PLoS One. 2025 Jun 4;20(6):e0323894. doi: 10.1371/journal.pone.0323894 (PMC12136332; doi:10.1371/journal.pone.0323894)
Supplement: Attachment — Submitted filename: respone-r2.pdf [file pone.0323894.s002.pdf]

Response to reviewers

We wish to thank the associate editor and the anonymous reviewers for their insightful comments and suggestions that led to improvements in the quality of the content and the presentation of our paper (*PONE-D-24-16152R1, "Dynamically weighted graph neural network for detection of early mild cognitive impairment "*).

The manuscript has been fully revised. Please find enclosed our comments on the reviewers' suggestions and comments and how we addressed and fixed all the comments and concerns raised.

Reviewer 3:

Comment：Thank you for addressing my concerns. I believe the manuscript, in its current state, is suitable for publication.

Response: Thank you for your feedback and consideration. We appreciate your comments and are glad that you find the manuscript suitable for publication.

Reviewer 2:

Comment 1: While some of the comments raised in my first review have been satisfactorily addressed, the related work section, however, provides a deficient analysis of existing GNN-based approaches for the classification of brain disorders such as [R1] Classification of developmental and brain disorders via graph convolutional aggregation; Cognitive Computation, 2023; and [R2] HI-GCN: a hierarchical graph convolution network for graph embedding learning of brain network and brain disorders prediction; Computers in Biology and Medicine, 2020.

Response: We appreciate the reviewer's valuable feedback. We have supplemented our analysis of the research landscape on GNNs for brain disorder classification, specifically referencing [R1] and [R2] to enrich the discussion and comparison of related work.

(1) Regarding [R1] (Classification of Developmental and Brain Disorders via Graph Convolutional Aggregation)：

This study proposes a graph convolutional aggregation (GCA) approach, incorporating skip connections and identity mapping to learn discriminative graph representations for the classification of autism spectrum disorder (ASD) and Alzheimer's disease (AD). In contrast, our approach utilizes a Dynamically Weighted GNN, which not only considers the static topology of the graph but also integrates temporal functional connectivity (FC) networks as weighted adjacency matrices in the GNN, allowing for a more comprehensive capture of the dynamic

characteristics of brain networks.

(2) Regarding [R2] (HI-GCN: A Hierarchical Graph Convolution Network for Graph Embedding Learning of Brain Network and Brain Disorders Prediction)

This study introduces a Hierarchical GCN (hi-GCN) framework, emphasizing multi-level feature learning and individual-population network fusion. hi-GCN enhances classification accuracy by integrating the topological information of brain networks with inter-subject correlations. In contrast, our method incorporates LSTM with Dynamic GNN, utilizing temporal FC modeling to learn the variations in brain functional connectivity over time, thereby improving eMCI classification performance.

We have incorporated the above comparative analysis into the Related Work section to highlight the novelty of our study and to more clearly position our contributions. Thank you for your suggestion!
